# Experiences of Pre-Exposure Prophylaxis Stigma, Social Support, and Information Dissemination Among Black and Latina Transgender Women Who Are Using Pre-Exposure Prophylaxis

**DOI:** 10.1089/trgh.2019.0014

**Published:** 2019-08-30

**Authors:** Ronald A. Brooks, Alejandra Cabral, Omar Nieto, Anne Fehrenbacher, Amanda Landrian

**Affiliations:** ^1^ Department of Family Medicine, University of California, Los Angeles, California.; ^2^ Center for HIV Identification, Prevention, and Treatment Services (CHIPTS), University of California, Los Angeles, California.; ^3^ Department of Community Health Sciences, Fielding School of Public Health, University of California, Los Angeles, California.; ^4^ Department of Psychiatry and Biobehavioral Sciences, Semel Institute, University of California, Los Angeles, California.

**Keywords:** Black, African American, Latina, transgender women, pre-exposure prophylaxis, stigma

## Abstract

**Purpose:** In the United States, HIV disproportionally affects Black and Latina transgender women (BLTW). Pre-exposure prophylaxis (PrEP) is a proven biomedical method for preventing HIV acquisition. However, the social stigma attached to using PrEP may deter uptake and persistence of PrEP among BLTW, two highly vulnerable populations. The purpose of this study was to explore the experiences of PrEP stigma among BLTW who are using PrEP in Los Angeles County.

**Methods:** In-depth, semistructured interviews were conducted with BLTW PrEP users (*N*=19) to explore experiences of anticipated, enacted, and internalized PrEP stigma within the context of their unique social and contextual factors. A thematic analysis approach was used in the analysis of qualitative data.

**Results:** We noted an underlying theme of HIV stigma related to participants' identification as trans women that served as the social context for other experiences. In addition, our data revealed five themes related to the experience of using PrEP. Three themes were specifically related to PrEP stigma and included: (1) Perception that BLTW PrEP users are HIV-positive; (2) perception that BLTW PrEP users engage in elevated sexual risk behaviors; and (3) negative labels ascribed to BLTW PrEP users. A fourth theme identified was the positive experiences of social support after PrEP disclosure reported by BLTW. Our fifth theme identified involved the dissemination of PrEP information by BLTW to friends/peers and sex partners.

**Conclusion:** BLTW experience PrEP stigma within the context of PrEP disclosure. This experience is underscored by existing experiences of HIV stigma connected to their identity as trans women. PrEP providers should prepare BLTW to use selective disclosure practices when revealing their PrEP use to help minimize experiences of PrEP stigma or potential physical harm. PrEP implementation programs should also support peer-to-peer PrEP education programs for transgender women to promote positive views of PrEP and help facilitate PrEP uptake in this population.

## Introduction

In the United States, transgender women (TW) remain a population disproportionately affected by HIV. The term “transgender woman” refers to a person whose gender identity is female/woman (or any other term along the trans feminine spectrum), but who was assigned male sex at birth.^1–3^ Although TW are underrepresented in HIV surveillance data,^4,5^ national estimates suggest that their rate of HIV infection is in the range of 22–28%,^6–8^ with Black and Latina transgender women (BLTW) experiencing the highest number of HIV diagnoses. Of the total number (*N*=1974) of TW who were diagnosed with HIV between 2009 and 2014, the Centers for Disease Control and Prevention (CDC) reports that 51% identified as Black, 29% identified as Latina, and 11% were white.^8^ The persistent HIV disparity experienced by BLTW underscores the need to increase uptake of new HIV prevention strategies with this highly impacted and vulnerable population.

One biomedical strategy for reducing HIV transmission among high-risk populations is with the use of pre-exposure prophylaxis (PrEP) (emtricitabine/tenofovir disoproxil fumarate). Data from efficacy trials indicate that when taken daily, PrEP is up to 99% effective at preventing the transmission of HIV in high-risk populations.^9–11^ The Pre-Exposure Prophylaxis Initiative (iPrEx) study was the only efficacy trial to enroll trans participants.^4,5,12^ A subanalysis of the TW enrolled in iPrEx (*N*=339) revealed that there were no HIV infections among TW who had PrEP drug concentrations that were commensurate with the suggested minimum dosing of four pills per week.^13^ Despite limited efficacy data on TW, PrEP remains a highly effective option for preventing the acquisition of HIV in this population. Unfortunately, disparities persist in the uptake of PrEP among TW.

According to recent CDC estimates, >1.1 million adults in the United States could benefit from the use of PrEP, yet <1% of those prescriptions were filled by eligible TW who could benefit from the use of the medication.^14,15^ Multiple studies have shown that in addition to the sexual risks they share with men who have sex with men (MSM) (e.g., condomless sex, multiple partners), social and contextual factors such as mental health issues, incidences of gender-based violence or transphobia, economic marginalization, incarceration, and higher rates of sex work contribute to the heightened HIV risk profile of TW.^6,13,16–18^ This suggests a need to tailor PrEP implementation efforts to address the barriers to PrEP uptake, adherence, and continuation experienced by TW, particularly BLTW.

Previous research has identified multiple barriers to PrEP uptake and continuation that are specific to the trans community. These include medical mistrust because of perceived or experienced transphobia^12,16,19^; a lack of access to gender-affirming health care^5,16,19^; concerns about the routine medical monitoring of PrEP and the management of multiple medications^16^; a higher prioritization for hormone therapy^16,20^; potential side effects or contraindications with hormone therapy^5,19,20^; lack of trans inclusive PrEP marketing^16,19,20^; and instability because of homelessness and economic marginalization.^5^ In addition, the intersection of HIV stigma and transphobia, particularly in the context of sex work, may also act as a deterrent to PrEP adoption among BLTW.^16,19,20^

The purpose of the study was to explore experiences of anticipated, enacted, and internalized PrEP stigma among BLTW PrEP users. Stigma is defined as a negatively valued and enduring attribute that marks an individual as socially deviant and functions to maintain differences in social equality between those experiencing stigma and those who do not.^21–23^ Stigma can be enacted, anticipated, and/or internalized.^22^ Enacted stigma refers to overt acts of discrimination, such as unfair treatment, social rejection, and violence; anticipated stigma deals with the expectation or belief that acts of prejudice and discrimination will likely occur in the future; and internalized stigma refers to the degree to which an individual endorses negative perceptions or feelings about themselves.^22^ An exploration of stigma among BLTW requires consideration of their unique social context.

## Methods

A purposive sample of BLTW PrEP users was recruited from October 2017 through November 2018 to complete an in-depth, semistructured qualitative interview about their experiences using PrEP. BLTW were eligible to participate if they were 18 years of age or older, identified as Black/African American or Latina/Hispanic, self-identified as a trans woman, had sex with a cisgender man in the last 6 months, were currently prescribed and taking Truvada for PrEP, had been using PrEP for at least 1 month, and resided in Los Angeles County. All eligible participants received $50 in gift cards (VISA or Target) for their participation.

Recruitment strategies included disseminating study promotional materials at trans-specific community events and at LGBT community agencies, and through participant referrals. Several barriers to recruitment of trans women observed in the study included loss of contact with potential participants because of a lost or stolen phone, incarceration, or housing instability. An additional barrier was PrEP discontinuation among BLTW PrEP users, which occurred between the initial contact about the study and the point of enrollment. Recruitment of participants was terminated when data saturation was reached with completed interviews (i.e., no new information was being gleaned from interviews).

A semistructured interview guide was developed to explore experiences of stigma related to the use of PrEP among BLTW. As part of the interview, participants were asked to describe the following: (1) experiences disclosing their PrEP use to friends/peers, family, sex partners, and medical providers; (2) experiences where they did not disclose their PrEP use because they thought they would be judged or treated differently; and (3) personal feelings related to their PrEP use. Interviews were audio recorded and lasted 60–90 min. All interviews were transcribed verbatim and checked for accuracy by two research staff members. A self-administered ACASI was used to gather quantitative data on participants' sociodemographic information, PrEP adherence and disclosure practices, and sexual behaviors. Each participant was assigned a unique participant identification number to maintain confidentiality. The Institution Review Board (University of California, Los Angeles) approved all study materials and all participants provided informed consent before the initiation of any study procedures.

The coding process is detailed in a previous article.^24^ In short, initial codes were developed from the interview guide, field notes, and multiple readings of the transcripts. Once consensus was reached on the codebook, a subset of codes was used to test for intercoder reliability. Two research team members independently coded two randomly selected transcripts and an intercoder reliability score was computed (Cohen's kappa coefficient, *k*=0.87). The final codes were entered into ATLAS.ti (version 8.3.20.0) and attached to their associated quotations for all transcripts. A thematic analysis approach was then used for analyzing all qualitative data.^25^ Coded data extracts were sorted into potential themes and reviewed by the study team to refine each theme. Major themes were selected based on their frequency across the dataset or the depth of the discussions.^25^

## Results

Demographic, PrEP use characteristics, and sexual behaviors are provided in Table 1. A total of 19 BLTW participated in the study, of which 10 identified as Black/African American and 9 identified as Hispanic/Latina. Overall, the median age was 28 (range=21–50). The majority identified as straight/heterosexual (68.4%), reported completing less than a college degree (57.9%), unemployed (42.1%), and having an annual household income of <$10,000 (63.2%). The majority of participants also had health insurance (84.2%) and reported being single (78.9%). The mean length of time on PrEP was 6.2 months (standard deviation=9.9; median=2.5; range=0.5–44.0). A little more than half (57.9%) of participants reported disclosing their PrEP use to at least a few people, and about three quarters (73.7%) of participants described their medication adherence as “very good” or “excellent.” Most participants reported using a condom during their last receptive or insertive anal sexual encounter with main, casual, or exchange male sex partners. Nearly half (47.3%) of participants reported having an exchange partner in the last 6 months.

**Table 1.** Demographics, Pre-Exposure Prophylaxis Use Characteristics, and Sexual Behaviors of Black and Latina Trans Women Pre-Exposure Prophylaxis Users (*N*=19)

Characteristic	*n* (%)
Demographics	
Age (in years)	M=31.47, SD=10.10
Racial/ethnic identity	
Black/African American	10 (52.6)
Hispanic/Latina	9 (47.4)
Sexual orientation	
Gay/homosexual/queer/same gender loving	4 (21.1)
Bisexual	2 (10.5)
Straight or heterosexual	13 (68.4)
Highest level of education completed	
7th grade	1 (5.3)
11th grade	3 (15.8)
High school graduate or received GED	7 (36.8)
Some college, AA degree, trade/technical school	6 (31.6)
Bachelor's degree (BA, BS)	2 (10.5)
Employment status	
Working full or part-time	7 (36.8)
On permanent disability	3 (15.8)
Unemployed	8 (42.1)
Other^a^	1 (5.3)
Annual income	
$0–9999	12 (63.2)
$10,000–19,999	3 (15.8)
$20,000–39,999	3 (15.8)
$40,000–59,999	1 (5.3)
Health insurance	
Does not have health insurance	3 (15.8)
Private medical insurance or employer-provided insurance	4 (21.1)
Medi-Cal/Medicaid or Medicare	12 (63.2)
Relationship status	
Single and not dating anyone special	15 (78.9)
Dating someone in an open relationship (have sex with other people)	1 (5.3)
Dating someone in a closed relationship (do not have sex with other people)	2 (10.5)
Partnered or married in an open relationship (have sex with other people)	1 (5.3)
PrEP use characteristics	
Length of time using PrEP (in months) (*N*=19)	M=6.18, SD=9.87
Number of people told about PrEP use	
No one	1 (5.3)
A few people	11 (57.9)
A lot of people	7 (36.8)
Disclosed PrEP use to (*n*=27)	
My main partner or spouse	3 (15.8)
One or more other sex partners	10 (52.6)
One or more family members	5 (26.3)
One or more friends	11 (57.9)
Health care providers	7 (36.8)
Other^a^	2 (10.5)
Adherence to PrEP medication past month^b^	
Fair	1 (5.3)
Good	4 (21.1)
Very good	6 (31.6)
Excellent	8 (42.1)
Sexual Behaviors by partner type (main, casual, exchange)	
Number of main^c^ male sex partners past 6 months (*n*=10)	M=3.1, SD=2.6
Number of times RA sex past 6 months (*n*=10)	M=27, SD=36.6
Last RA sex encounter condoms used	
Yes	6 (60.0)
No	4 (40.0)
Number of times IA sex past 6 months (*n*=10)	M=6.6, SD=11.2
Last IA sex encounter condoms used (*n*=5)^d^	
Yes	4 (80.0)
No	1 (20.0)
Number of casual^e^ male sex partners past 6 months (*n*=13)	M=11.9, SD=13.2
Number of times RA sex past 6 months (*n*=13)	M=14, SD=13.9
Last RA sex encounter condoms used (*n*=12)^d^	
Yes	9 (75.0)
No	3 (25.0)
Number of times IA sex past 6 months (*n*=13)	M=4.7, SD=6.2
Last IA sex encounter condoms used (*n*=10)^d^	
Yes	6 (60.0)
No	4 (40.0)
Had sex with an exchange partner past 6 months	9 (47.3)
Number of exchange^f^ male sex partners past 6 months (*n*=9)	M=27.7, SD=29.5
Number of times RA sex past 6 months (*n*=9)	M=10.3, SD=6.7
Last RA sex encounter condoms used	
Yes	6 (66.7)
No	3 (33.3)
Number of times IA sex past 6 months (*n*=9)	M=7.4, SD=6.5
Last IA sex encounter condoms used (*n*=7)^d^	
Yes	5 (71.4)
No	2 (10.5)

^a^ Others not specified.

^b^ PrEP adherence was measured through self-report using a validated Likert scale from very poor to excellent.^39^

^c^ Main partner refers to someone with whom the participant has a close, ongoing, intimate relationship with.

^d^ Excludes participants who indicated never using a condom in the past 6 months.

^e^ Casual partner refers to someone with whom the participant has sex with, but do not consider a main or steady partner.

^f^ Exchange partner refers to someone with whom the participant has sex with in exchange for things they need such as money, drugs, shelter, or food, and who are not considered main or casual partners.

GED, General Education Development; IA, insertive anal; M, mean; PrEP, pre-exposure prophylaxis; RA, receptive anal; SD, standard deviation.

In describing their experiences using PrEP, participants reported an underlying experience of HIV stigma related to their identity as a transgender woman. Participants shared that family members and those within the cisgender community, particularly cis women, hold the beliefs that all TW will contract HIV in their lifetime, are already HIV-positive, and are to blame for the continuing spread of HIV (Table 2, Quotes 1–3). This form of stigma manifested as public verbal attacks, death wishes, judgment, or rejection (Quotes 1–3). These experiences provided the background for understanding reported instances of PrEP stigma.

**Table 2.** Experiences of HIV Stigma Related to the Identity of Transgender Women

Enacted stigma
(1) They look at me because I'm transgender, many think, automatically, that we're [HIV-positive]… Like I told you about that incident where I was attacked, the women said, “You fucken’ HIV-infected, *bitch*!” Right away, that stigma and that really burned me up not only because I know I'm not, but that was the true picture of how a lot of cisgender women of color view us, as women giving people HIV. (Latina, age 50, 1 month on PrEP)
(2) I just feel like people are in such a frame of mind where they think that HIV is just centered in one area and [not in the] cisgender community, in the women community. It's like, “It's okay for you to talk to me about my HIV status, but are you talking to every partner that you come in contact with the way you're talking to me because I'm trans?” […] If you can ask me about my HIV status and I can report to you and send you my result card, I'm expecting you to be able to do the same thing. (Black, age 48, 6 months on PrEP)
(3) When I was younger, a lot of people would say that… because a lot of them are religious, they would say that I'm going to get AIDS and all type of stuff like that. So I purposely let my mom know that I don't have it and that I'm taking something that will prevent me from getting it… Because my mother was always the one who told me that I'm not going to be nothing in life, and she hopes I die, and if she dies, she doesn't want me to come to the funeral. She always had so much mean stuff to say. She even said one time, “I'm sure you're going to get AIDS before you turn 21.” (Black, age 29, 7.5 months on PrEP)

Our data revealed five themes related to the experience of using PrEP. Three themes were related to PrEP stigma: (1) perception that BLTW PrEP users are HIV-positive; (2) perception that BLTW PrEP users engage in elevated sexual risk behaviors; and (3) negative labels ascribed to BLTW PrEP users. The prevailing sources of anticipated and enacted stigma included family, peers/friends, sex partners, and those within the cisgender community. In addition, we identified a robust theme related to social support of PrEP use among BLTW. Sources of social support included peers/friends, family, and sex partners. A related theme involved the dissemination of PrEP information to trans peers and sex partners.

### Perception that BLTW PrEP users are HIV-positive

A prominent theme related to PrEP stigma was the assumption that BLTW PrEP users are HIV-positive because they are taking an HIV medication. This resulted in other trans peers discrediting the individual because of their presumed HIV-positive status (Table 3, Quote 1). Participants also described potential questions that may arise about their HIV status if they were to disclose their PrEP use (Quotes 2–3, 6). As a result, some participants opt to not disclose their PrEP use (Quotes 2, 6) or conceal their PrEP pill bottles (Quote 4), which allowed for more agency and control over disclosure. One participant was counseled by her doctor not to reveal her PrEP use for safety reasons (Quote 5). Other participants were motivated to conceal their PrEP use because of concerns of physical harm or violence should their cisgender male partners discover their use of PrEP (Quote 6). In stark contrast, some PrEP disclosure events provided sex partners with proof of the person's HIV-negative status (Quote 7).

**Table 3.** Perception that Black and Latina Trans Women Pre-Exposure Prophylaxis Users Are HIV-Positive

Enacted stigma
(1) My friends when they found out were like, “Oh, girl… you're infected! You're HIV-positive. Bitch, get away from me! That bitch is taking Truvada. You guys better watch out and you better get yourself tested.” […] So I said, “Since I'm taking Truvada, I'm not like the rest of you bitches trying to get all these little tricks infected with HIV and not knowing what the fuck you're doing.” (Latina, age 25, 3 months on PrEP)
Anticipated stigma
(2) I think my family would react in a way that they would think I probably have HIV, and that that's why I'm taking it. Because they're not as informed on things like that, so they'll be like, “Why are you taking it? Do you have it already?” Because it's called Truvada—isn't that the same name for the medication for the people that have HIV? (Latina, age 21, 1.75 months on PrEP)
(3) To be honest with you, most of the sexual partners that I have sex with are heterosexual men. So therefore when you tell them PrEP, they're like, “What's that?” And then I don't want to go explain that it's an HIV med because then they're going to start thinking, “Oh, you're HIV-positive.” (Latina, age 42, 6 months on PrEP)
(4) Maybe if I'm meeting a guy for the first time and he's coming over to my place, I would probably hide it because he could mistake it for an HIV medication. And I would like to have a conversation with him before, and I would—I *would* have a conversation with him the first time and tell him about my PrEP usage. (Latina, age 21, 1.75 months on PrEP)
(5) [My doctor] told me, he says, “Better yet, don't let people know what kind of meds you're on… but if a person does go into your medical cabinet and they see that [PrEP], then they're going to question you, for safety concerns, because you're transgender.” […] To me, that was a plus to care about our safety because people that we sleep with [heterosexual, bisexual, or gay men]… when they know what it's about…“Well, hold on bitch! You have it… you're trying to infect me.” So that's why I said I put my medications away because it's nobody's business. (Latina, age 50, 1 month on PrEP)
(6) [I don't disclose to some of my sexual partners] because there's really no strings attached, and the last thing I would want is to say something and somebody think I said something that I didn't or hear something that they didn't hear. I don't want to say, “Oh, I'm taking this.” And they're like, “What's that?” “Oh, it's an HIV pill.” All of a sudden they just hear “HIV” and they just blackout or hurt me. I always read the people that I'm with before I decide, and when I see that they're comfortable and I'm comfortable, then I'll disclose. (Black, age 29, 7.5 months on PrEP)
PrEP use a proof of HIV-negative status
(7) [My sex partners] are a little more relieved. I don't know if it's because they know you have to be negative to be on it, but they're more like, “Okay that makes me feel better to know that you don't have it.” (Latina, age 21, 44 months on PrEP)

### Perception the BLTW PrEP users engage in elevated sexual risk behaviors

Another theme that emerged was the perception that BLTW PrEP users are more likely or willing to engage in risky sexual behaviors. This perception led some to express internalized feelings of guilt for continuing to engage in behaviors that put them at increased risk for contracting HIV (e.g., condomless sex and multiple partners) (Table 4, Quote 1). The belief that PrEP users do not have to use condoms or are more willing to have condomless sex also made it difficult for some participants to negotiate condom use with sex partners (Quotes 2–3).

**Table 4.** Perception That Pre-Exposure Prophylaxis Users Engage in Elevated Sexual Risk Behavior

Internalized stigma
(1) I've just been kind of guilty because I sometimes don't use condoms and I should be using more condoms. That's why I'm having these classes where I'm learning more about causes of HIV and all that. Sometimes I do feel like, damn, why didn't I just use condoms? I should have, but of course it feels better without a condom—it's really tempting. Like yesterday, I didn't use a condom. It worries me sometimes, but, at the same time, I'm in the safe side—hopefully a safe side. (Latina, age 23, 2.5 months on PrEP)
Enacted stigma
(2) Since I've been on it, I really haven't had too much sex because it's just my choice right now, but when I tell people that I'm on it, the ones that I've had sex with, keeping it real, a lot of them still ask, “Well, why do I have to use a condom?” So I tell them, “Because you don't know what I might have.” They don't understand. (Latina, age 50, 1 month on PrEP)
(3) And then the people in the dating apps, they are very confident about it. They want to have unprotected sex all the time. I'm like, “No, I don't want that.” […]When they tell me that they are using it, I feel more safe. But, sometimes, they go for unprotected sex and they insist when they know I am. I'm like, “No.” (Latina, age 23, 14 months on PrEP)

### Negative labels ascribed to BLTW PrEP users

A related theme was the experience of being assigned a negative label or identity because of their PrEP use. This included the attachment of negative labels such as “whore” or “slut” (Table 5, Quotes 1–2). Sources of labels were friends and ex-partners.

**Table 5.** Negative Labels Ascribed to Black and Latina Trans Women Pre-Exposure Prophylaxis Users

Enacted stigma
(1) I told [my ex-boyfriend] and he was like, “Why are you drinking that?” We were not on anything by then, but he was like crazy stalking me, spying on me. I would talk to him and I told him that I was using this. His response was like, “You are going to be a whore. That's why you want it for.” I really wanted it for that boyfriend who was HIV-positive, but he didn't know about it. (Latina, age 23, 14 months on PrEP)
(2) [My friend] said that I was a slut. I was like, “But I'm a protected slut bitch! I'm putting up barriers.” (Black, age 48, 2.5 months on PrEP)

### Social support after PrEP disclosure

Despite a negative reception after PrEP disclosure for some participants, others reported receiving positive social support and encouragement from friends and family, sex partners, and peers within the trans community for taking precautions to prevent HIV infection (Table 6, Quotes 1–3). For TW who engage in sex work, much of this support originated from the mutual understanding that PrEP is a necessary form of protection given their line of work (Quotes 2–3).

**Table 6.** Social Support Following Pre-Exposure Prophylaxis Disclosure

(1) [My friends] were happy. They were excited for me. I've always been warned, “Don't trust anybody.” Now that I'm on PrEP, they're happy for my health status, that I won't catch the virus if a condom breaks or just taking that risk. (Latina, age 50, 1 month on PrEP)
(2) It was a good thing; [my parents] recommended it [PrEP]… They think it's the best for me because a girl like me should always be safe with my sex life as well, regardless of who I'm having sex with. So they always say, “Think about that before you end up having sex with the same person or sleeping with somebody in that bed with you and not knowing their status.” (Black, age 28, 0.5 months on PrEP)
(3) Well, my mom knows what I have to do to make my ends-meet out here, so she definitely encourages it… A lot of [the support I received] was positive, given the type of work that I've had to resort to. Again, this is coming from the trans community, so they're a lot more accepting. So friends have always been supportive about it. Other than that, I haven't had much backlash with friends. (Latina, age 21, 44 months on PrEP)

### Dissemination of PrEP information

To help reduce HIV transmission in the trans community, some participants used their disclosure event as an opportunity to disseminate information about PrEP to other TW, particularly those who engage in sex work (Table 7, Quotes 1–3). Participants also disseminated information to heterosexual male partners who do not perceive themselves to be at risk for HIV (Quote 3).

**Table 7.** Dissemination of Pre-Exposure Prophylaxis Information

(1) Most of the trans women that I talked to, they do the same thing I do. So therefore, they're like, “Oh, really?” They wanted to try it to see if they can do it. (Latina, age 42, 6 months on PrEP)
(2) I think that it's very important to share this information with all people of color—well, not just people of color, but with everybody…. A lot of my girls are into the sex work. They make more money without the condom than with the condoms. So for them, it's really like, “Yes, it's for you. You need to be on it so that way you can be protecting yourself because it's not like sex work is all that we can do now.” And I don't take anything away from anyone who does sex work—they got to make their money too—but if you're going to be out there making your money, at least make it in a way that you can sleep good at night and know that you have some form of protection in your body against these people that you know nothing about. (Black, age 48, 6 months on PrEP)
(3) I haven't had that many [sex partners] recently, but I do let them know, “Look, I don't have HIV and I'm on PrEP. What do you want to do?” And I even ask if they know about it. I've had the opportunity to disseminate the information right before we decide to have sex. Like, I get to have a nice conversation about why it's important. Even him, as a *straight* man, should be on PrEP. (Black, age 48, 2.5 months on PrEP)

## Discussion

Our study sample consisted of BLTW who experience multiple vulnerabilities, as evidenced by high levels of unemployment, low annual incomes, and a high percentage currently engaged in transactional sex (i.e., sex work). Nonetheless, participants reported high levels of medication adherence. When disclosing their PrEP use, BLTW were met with both negative and positive responses from friends/peers, family members, and sex partners. Negative responses commonly included the assumption that BLTW are HIV-positive and/or engage in risky sexual behaviors, and resulted in others labeling them as hypersexual (e.g., “slut” or “whore”). These findings are similar to what have been reported among MSM PrEP using populations.^24,26–28^ More nuanced experiences of PrEP stigma included the discrediting of BLTW PrEP users by other trans peers and cis women, and difficulty in negotiating condom use with sex partners or clients. Previous research has shown that TW report higher rates of condomless sex with primary sex partners, low self-efficacy in their ability to effectively use condoms, and reduced power in negotiating condom use because of fear of retaliation after a request for safer sex.^29–31^ Once it is known that they are on PrEP, BLTW may experience an additional barrier in negotiating condom use with their partners because of the assumption that PrEP users prefer to engage in condomless sex. The following discussion provides a further interpretation of the experiences of PrEP stigma in light of the unique social and contextual factors of BLTW.

This study revealed an underlying experience of HIV stigma directly related to the identity of TW, such as the belief that all TW are vectors of HIV infection. This finding is supported by previous research.^16,32^ With regard to their PrEP use, BLTW were also confronted with the assumption that they are HIV-positive and trying to conceal their status. In certain situations, this assumption may carry with it the potential for physical harm or intimate partner violence (IPV). For example, HIV stigma is often magnified within the context of sex work, which manifests in the individual's presumed HIV-positive status being leveraged against them by other trans peers to limit their ability to secure work and in the potential for retaliatory violence from clients.^16,20,32^ The social stigmas attached to PrEP may therefore act as a barrier to PrEP uptake among vulnerable BLTW, who may wish to avoid further marginalization or harm should their PrEP use be discovered.^16,20,26,32,33^ PrEP implementation programs will need to address multiple stigmas (HIV, PrEP, and transphobia) to successfully support the adoption and continuation of PrEP among BLTW.

While an outlier supported by the experience of one participant, medical providers emerged as an important source of information for TW regarding the potential harm that may result if their PrEP use is discovered and others assume she is HIV-positive. We believe that health care providers who deliver PrEP should be prepared to assist BLTW with developing adaptive strategies to mitigate experiences of stigma and IPV associated with their PrEP use (e.g., skills building around selective disclosure practices, hiding PrEP pill bottles, removing labels, and using pill boxes). These types of supportive services should be embedded within the delivery of PrEP to BLTW.

Another important finding in this study was the social support BLTW PrEP users received regarding their use of PrEP. Sources of encouragement and praise included family members, friends, and trans peers. In particular, the support participants received from family in their initiation of PrEP was a unique finding given that TW have typically reported hostility (e.g., misgendering), rejection, and a lack of social support from their family of origin.^34,35^ Conversely, Seibel et al.^36^ posit that TW who report increased familial support experience higher self-esteem and advancement of their gender confirmation. Although not fully explored in this study, or with the authors cited previously, these findings suggest that family support may play an instrumental role in helping to facilitate PrEP adoption among at-risk BLTW and in improving their overall mental and emotional well-being. In addition, in this study we found trans peers were particularly supportive of participants' PrEP use, especially in relation to their shared experiences of sex work. Social support within the trans community may help lessen the effects of PrEP stigma much in the same way that it has done with other forms of stigma.^1,37,38^ Social support in the context of PrEP use may also help facilitate PrEP uptake and persistence among highly marginalized BLTW.^37^

Sharing information about PrEP with peers is another way to help foster PrEP uptake in marginalized populations. In this study, BLTW described experiences of disseminating PrEP information to their peers who are at a high risk for HIV infection. This finding aligns with other literature suggesting that peer education may be an appropriate model for disseminating information about PrEP to high-risk populations, including trans populations.^16^ We believe that peer educators can support the adoption of PrEP among BLTW by promoting a more positive social view of PrEP and highlighting the benefits for individuals and the community. This would include dispelling common myths about PrEP found among TW (e.g., that there are contraindications with PrEP and feminizing hormone therapy), which is a recognized barrier to PrEP uptake in this population.^16,20^

## Limitations

Interpretation of these findings should take into account our study limitations. We recruited a convenience sample of BLTW PrEP users in Los Angeles, and results may not reflect the experiences of BLTW PrEP users in other geographic locations. In addition, the sample consists exclusively of English-speaking Latina TW and may not reflect the experiences of Spanish-speaking Latina TW. Research with monolingual Spanish-speaking Latina TW is needed to assess if experiences of PrEP stigma differ for Spanish speakers, particularly for undocumented TW. A potential bias in our sample is that the study population included BLTW who had been on PrEP, on average, for only 6 months, and who may still be adapting to the experiences of PrEP stigma. Future research should include BLTW PrEP users who are long-term users (e.g., a year or more) to assess if long-term use of PrEP heightens or lessens experiences of PrEP stigma and its personal and social consequences.

## Conclusion

Despite these limitations, the study has important implications for mitigating PrEP stigma in the context of disclosure and supporting uptake and continuation of PrEP among BLTW. Given the vulnerability and victimization experienced by some BLTW, there is a need for BLTW PrEP users to possess the skills needed to assess the potential consequences of each disclosure event. We believe that medical staff working with this population need to understand the unique social and contextual factors of BLTW to provide appropriate support to them in their use of PrEP. Our findings also highlighted the importance of peers in supporting adoption and disseminating PrEP information. This suggests to us that peer educators could help in deconstructing the existing PrEP stigma and promoting a positive view of PrEP users within the trans community.

## Abbreviations Used

ACASI: Audio Computer-Assisted Self-Interview
BLTW: Black and Latina transgender women
CDC: Centers for Disease Control and Prevention
GED: General Education Development
IA: insertive anal
iPrEx: Pre-Exposure Prophylaxis Initiative
IPV: intimate partner violence
MSM: men who have sex with men
PrEP: pre-exposure prophylaxis
RA: receptive anal
TW: transgender women
